# Functional Treatment of a Child with Extracapsular Mandibular Fracture

**DOI:** 10.1155/2017/9760789

**Published:** 2017-05-24

**Authors:** Diana Cassi, Marisabel Magnifico, Chiara Di Blasio, Mauro Gandolfini, Alberto Di Blasio

**Affiliations:** ^1^Section of Orthodontics, University Dental Center, Department of Biomedical, Biotechnological and Translational Sciences, University of Parma, Parma, Italy; ^2^Maxillofacial Surgery Division, General and Specialist Surgery Department, University Hospital of Parma, Parma, Italy

## Abstract

Condylar fractures are among the most frequent fractures in the context of traumatic lesions of the face. The management of condylar fractures is still controversial, especially when fractures occur in children: if overlooked or inappropriately treated, these lesions may lead to severe sequelae, both cosmetic and functional. The therapy must be careful because severe long-term complications can occur. In this case report, the authors present a case of mandibular fracture in which the decision between surgical therapy and functional therapeutic regimen may be controversial due to the particular anatomy of the fracture line and the age of the patient.

## 1. Introduction

Fractures of the mandible represent a frequent accident, being 11–16% of all facial fractures [1–3]. Notably, about 30–40% of mandibular fractures involve the condyle [4–6]. Fractures of the mandibular body are generally caused by direct trauma, whereas most of the condylar injuries are the result of indirect forces, usually applied to the chin. Owing to the few symptoms and the inadequate radiographic examination, mandibular condylar fractures (MCF) are frequently undiagnosed. The orthopantomography was considered for a long time the ideal examination, but it has now been replaced by the CT scan because sometimes only 3D imaging allows identifying the problem [7–10]. Depending on the anatomical level of the fracture, MCF may be divided into intracapsular, involving the condylar head, and extracapsular regarding the condylar neck or the subcondylar region [8]. However, the term intracapsular is not accepted by all authors because the fracture line, starting in an intracapsular position, often drops outside in the extracapsular area; therefore, the term “diacapitular fracture” (DF) has been proposed to better describe this condition [11–15]. The management of condylar fractures is still controversial. The treatment approach includes (A) surgical open reduction and internal fixation (ORIF) [16, 17] or (B) closed functional therapeutic regimen (CTR) [18, 19].

As stated in 2012 by Chrcanovic [11], the current indications for the ORIF are (1) fractures involving the lateral aspect of the condyle associated with reduction of mandibular height and (2) fractures in which the cranial fragment dislocates laterally out of the glenoid fossa. On the contrary, the functional treatment is generally preferred in children and is recommended for fractures without displacement of fragments or when the displacement involves the medial parts of the condyle without shortening of the condylar height.

Condylar fractures in the pediatric age occur on a rapidly growing bone: if overlooked or inappropriately treated, these lesions may lead to severe sequelae, both cosmetic and functional. The therapy must be careful even because severe long-term complications can occur. The most dangerous complication is real ankylosis of the temporomandibular joint (TMJ), with reduced mandibular function and restricted mouth opening, chronic pain, and loss of ramus height; class II malocclusion with anterior open bite may also occur [1–6, 20, 21].

Although pediatric facial traumatology is the most common cause of pathological changes in TMJ, other conditions may reduce the mandibular mobility during the developmental age leading to severe TMJ disorders [20] and sometimes requiring complex surgical procedures.

In the functional ankylosis, the joint space becomes filled with a thick “organizing” tissue difficult to remove, with a progressive reduction of mandibular mobility. Generally, the recovery of oral functions is complete in children treated by CTR, although the condylar remodeling may be not be entirely satisfactory from a radiological point of view [7, 22]. Especially in subjects above 12 years old, even if the function is restored, the anatomy of the mandibular condyle may become improved but not completely corrected [2]. Thus, at about this age, the treatment of the patient should be considered similar to those directed to adults [22–24]. The cranial fragment undergoes resorption and the caudal fragment progressively regenerates, although the condylar remodeling to the original morphology can only be expected in children, not in adolescents or adults [23, 24]. Especially in situations in which the cranial fragment is lost in a growing patient, a complete recovery of oral functions is mandatory to ensure a further normal growth of the mandible [24]. In monolateral fractures, the risk consists in a unilateral reduction in mandibular growth, which in an advanced age may require complex orthodontic [25] or surgical procedures. In bilateral fractures, a severe class II may occur due to mandibular defect, leading to both functional and esthetic discomfort [26].

In this case report, the authors present a case of mandibular fracture in which the decision between ORIF and CRT may be controversial due to the particular anatomy of the fracture line and the age of the patient.

## 2. Case Report

**Figure 1:**
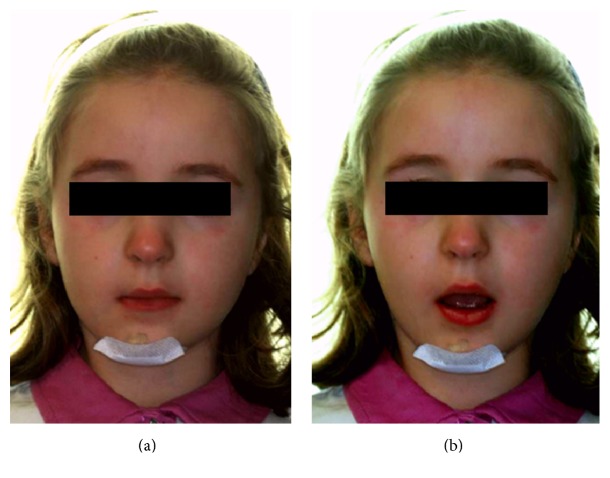
(a, b) Frontal view of the face and limitation in mouth opening.

A six-year-old girl was referred for a facial trauma to the UOC of Odontostomatology at University Hospital of Parma (Italy). The patient presented with a minor skin lesion in the chin area, only requiring a superficial medication (Figure 1(a)). She was affected by a mild pain and limitation in mouth opening (Figure 1(b)), without any problems in general health condition.

**Figure 2:**
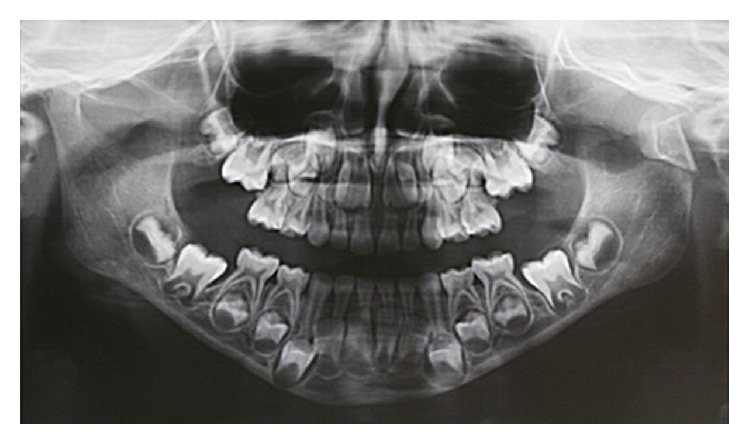
The large fracture area between the ramus and condylar neck.

An orthopantomography was taken as the first diagnostic imaging and the fracture line was clearly identified, running outside the capsular area and involving the condylar neck at the edge between the neck and the mandibular ramus. Despite the largely affected area, the vertical dimension and the occlusion were preserved and the cranial piece of the fracture was not widely dislocated from the caudal one, probably due to the integrity of the periosteal layer (Figure 2).

As a treatment solution, the orthodontist, together with the maxillofacial surgeon, decided to avoid the ORIF approach in favor of a modified CRT sequence.

The caudal fragment ensures insertion for the masseter and temporalis muscle, while the cranial fragment ensures insertion for the lateral pterygoid muscle. In this condition, early intense mobilization, as prescribed in the classic CRT, may cause further displacement of the cranial fragment [27–29]. Accordingly, a modified CRT sequence was performed, consisting in a delayed treatment with full functional exercises regimen, in order to allow the fibrous callus formation.

Interestingly, the functional therapy was not adopted to permit regeneration of condylar head and bone remodeling, but to maintain the functional integrity of the joint during the growth.

**Figure 3:**
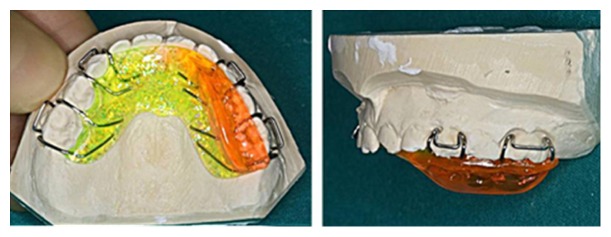
Functional removable appliance.

We first prescribed a week of functional minimal activity, soft diet, and FANS when needed for pain control. At the end of the first week, it was decided to start a modified CRT sequence for another week. Such sequence consists in the same functional exercises as in the classic CRT, but performed in a mild way. The patient was advised to move the mandible slowly, to avoid any pain, and to not try to improve the magnitude of the movement. After this phase promoting osseous union, the classic functional therapy was prescribed, including both full exercises and functional removable appliance (Figure 3).

**Figure 4:**
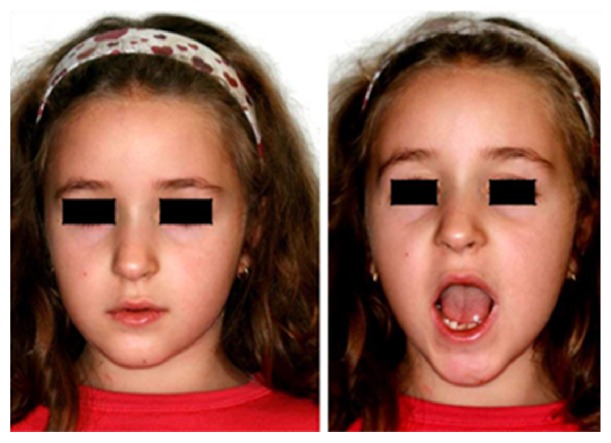
The good functional result of the therapy.

**Figure 5:**
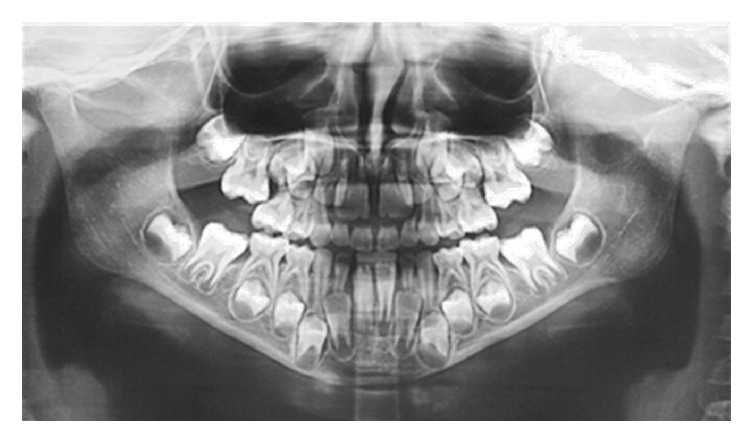
The complete anatomical restoring of the fracture.

The functional appliance maintained the mandible in a therapeutic position in protrusion and contralateral deviation and was prescribed by night. The series of functional exercises was suggested for 15 minutes, four times a day. The prescribed functional exercises were (A) maximum mouth opening, (B) maximum protrusive movement, and (C) maximum right and left lateral movements. The extension of these exercises was prescribed to the limit of the pain, maintaining the movement symmetry and trying to improve the range day by day. The modified CRT sequence was carried on for six months with good results in terms of jaw mobility (Figure 4) and a radiographic control was performed. In the new orthopantomography, the two fragments appeared perfectly jointed and the fracture line was no more visible (Figure 5).

**Figure 6:**
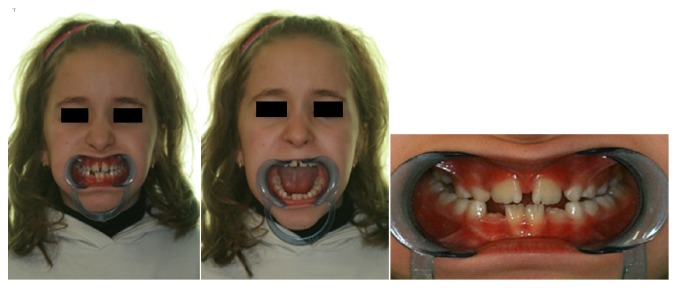
Functional results and frontal occlusion.

The removable functional appliance was then interrupted and the functional exercises were continued for a further period of six months (Figure 6).

## 3. Discussion

Conservative approaches in treating condylar fracture include physiotherapy, intermaxillary fixation (IMF) [18, 30], and functional appliances (e.g., activator) [31].

Temporary intermaxillary fixation (IMF) can be used in association with the functional treatment of pediatric mandibular condylar fractures. The IMF is applied for a short period followed by the use of orthodontic guiding elastics, which is used to guide the mandible into central occlusion. The most common methods are arch bars, eyelet wires, orthodontic brackets, vacuum-formed splint, using the teeth as the anchors to apply IMF, and screw-based appliances [18].

Some surgeons have found no benefit in the use of IMF saying that early mobilization of the mandible can improve vascular and lymphatic circulation adjacent to the fracture site and thus accelerate regeneration of the fractured condyle [21]. Moreover, IMF presents many disadvantages: deterioration in oral hygiene, tooth decay, injury to the dentition by fixation methods, malnutrition, and weight loss. It is also reported that longer periods of IMF can lead to bony ankylosis or fibrosis and severely limited mouth opening. For children, the treatment of condylar fractures with IMF is complicated by poor patient compliance, difficulty in applying IMF, and, in the case of mixed dentition, lack of sufficient support [19].

Functional appliances allow the restoration of a plane of occlusion orthogonally aligned to the forces of occlusion and a correct transfer of forces through the maxilla to the rest of the cranial bones, essential to allow proper facial development [21]. The principal aim of this approach is the activation of the bone remodeling process, the rebalancing of intra-articular functional structures, and the reacquisition of mandibular movements at the level of fracture condyle. This is accomplished through the early restoration of a stable occlusion and the normalization of the muscle functionality. Early joint activation also prevents functional limitations or ankyloses. Functional appliances have the advantage of being removable and well tolerated; however, they are limited by the patient's collaboration capabilities.

According to the scientific literature, the CRT approach is recommended for children with intracapsular mandibular fractures. In the authors' opinion, employing the CRT may also be considered for other particular situations in which the fracture line drops far from the condyle in an extracapsular position. In such cases, the following conditions are required in order to avoid the ORIF:

1. The two fragments are separated but not widely dislocated. This finding suggests that the periosteal layer is not interrupted, ensuring the contiguity of the bony pieces.
2. The fracture line does not involve the intracapsular area. This finding is fundamental because it ensures the absence of blood in the articular space. The absence of intra-articular blood avoids the risk of fibrous organization in the TMJ. For this reason, a two-week delay in starting the CRT may not be dangerous.
3. The vertical dimension and the occlusion are maintained.
4. The patient is of young age at the time of injury.

When these conditions occur, the authors suggest performing functional rehabilitation as previously described.

The main objectives of this approach are to restore integrity of TMJ function and normalize functional movements, avoiding neuromuscular adaptation. A gentle and early mobilization of the jaw does not prevent the fibrous union of the fractured fragments and helps the patients to achieve the pretraumatic range of motion.

A careful monitoring of recovery of mandibular movements and a radiographic control are mandatory in order to prevent resorption in favor of complete restoring of articular integrity. Long-term follow-up is necessary, as in all traumatic pathologies.
